# Biochar and biofertilizer reduced nitrogen input and increased soybean yield in the maize soybean relay strip intercropping system

**DOI:** 10.1186/s12870-023-04058-5

**Published:** 2023-01-17

**Authors:** Kai Luo, Chen Xie, Xiaoting Yuan, Shanshan Liu, Ping Chen, Qing Du, Benchuan Zheng, Zhidan Fu, Xiaochun Wang, Taiwen Yong, Wenyu Yang

**Affiliations:** 1 College of Agronomy, Sichuan Agricultural University, Chengdu, 611130 China; 2 Sichuan Engineering Research Center for Crop Strip Intercropping System / Key Laboratory of Crop Ecophysiology and Farming System in Southwest, Ministry of Agriculture, Chengdu, 611130 China

**Keywords:** Biochar (BC), Biofertilizer (BF), Nitrogen fixation, Soil properties, Soybean yield, Maize soybean relay strip intercropping

## Abstract

Applying Biochar (BC) or biofertilizers (BF) are potential approaches to reduce the nitrogen input and mitigate soil degradation in the maize soybean relay strip intercropping system (IS). In 2019 and 2020, a two-factor experiment was carried out to examine the effects of BC and BF on soil productivity and yield production in IS. 4 N input levels (8.4, 22.5, 45 kg, and 67.5 kg ha ^− 1^) referred to as N0, N1, N2, and N3 were paired with various organic treatments, including BC (150 kg ha ^− 1^), BF (300 kg ha ^− 1^), and without organic amendments (CK). The results demonstrated that, despite BF decreasing the biomass and N distribution into grains, BF performed better on improved soybean yield (5.2–8.5%) by increasing the accumulation of soybean biomass (7.2 ~ 11.6%) and N (7.7%). Even though BC and BF have a detrimental effect on soybean nitrogen fixation by reducing nodule number and weight, the values of soybean nitrogenase activity and nitrogen fixation potential in BF were higher than those in BC. Additionally, BF performs better at boosting the soil’s nitrogen content and nitrate reductase and urease activity. BF increased the concentration of total N, soil organic matter, Olsen-phosphorus, and alkaline hydrolyzable N in the soil by 13.0, 17.1, 22.0, and 7.4%, respectively, compared to CK. Above all, applying BF combination with N2 (45 kg ha ^− 1^ N) is a feasible strategy to raise crop grain output and keep soil productivity over the long term in IS.

## Introduction

Population growth widens the supply and demand gap, severely compromising food security [1]. As a result, boosting the output of grain, oil, and protein crops is crucial to resolving major food security issues by meeting daily dietary energy needs [2]. While intensive agriculture can enhance yield production by increasing the input of chemical fertilizer, the environmental harm and decreased soil productivity brought on by the misuse of chemical fertilizer have adversely affected stable long-term high production and human health [3–5]. Thus, limiting the chemical fertilizer input, especially nitrogen (N), in a sustainable agriculture system is crucial to decreasing environmental costs and maintaining long-term soil productivity [6]. A possible strategy to balance the high-yield output and minimal resource input is the intercropping system, in which two or more crops are planted on the same farmland [7].

The intercropping system, particularly the legume-cereal intercropping system, could provide high yield advantages [8], highly efficient resources usage [9], and high nutrient use efficiency [10]. Additionally, the maize soybean relay strip intercropping system (IS) fully utilizes soybean biological nitrogen fixation (BNF) to reduce nitrogen input and environmental contamination [11]. According to prior studies, using 270 kg ha^− 1^ N for IS might maximize N usage efficiency and achieve the balance between lower N input and higher yield output [12]. Meanwhile, the use of organic amendments (OA) [13] and rhizobium inoculants [14] are two promising solutions to reduce the input of chemical fertilizer to prevent soil deterioration in IS.

Applying OA in the intensive agriculture system is an age-old but gaining popular approach for boosting yields and preserving soil fertility [13]. Commonly utilized in some crops, including maize [15], soybean [16], and other crops [17], are biochar (BC) and biofertilizer (BF). In low-fertility soils, long-term application of BC and BF with appropriate N input is a great way to boost crop yields immediately and keep sustainable crop production going [18, 19]. Whether BC or BF can lower the N input and increase grain yield in IS is still unknown. Investigating a suitable combination of organic amendments and nitrogen fertilizer is crucial to reducing nitrogen input and increasing crop production in IS.

Thus, this experiment examines the impacts of BC and BF under various N inputs on soybean biological nitrogen fixation, yield formation, and soil characteristics in the maize soybean relay strip intercropping system. 1) How do organic materials affect the development of soybean nodules and biological nitrogen fixation under various N inputs? 2) Whether the combination of BC or BF with less N input could maintain soil characteristics and soybean yield production.

## Material and methods

### Plant material and growth conditions

A field experiment was carried out in 2019 and 2020 at Chong’zhou Modern Agricultural Research and Development Station (30° 56′N, 103° 64′ E), Sichuan Province, China. The field soil was a light loam with pH 6.99, 32.44 g kg ^− 1^ organic matter, 1.37 g kg ^− 1^ total N, 22.46 g kg ^− 1^, Olsen- phosphorus (Olsen-P), and 138 g kg ^− 1^ exchangeable potassium (K). In this experiment, shade-tolerant soybean ‘Nandou 25’ and compact maize ‘Denghai 605’ were employed.

The companies Liaoning Golden Future Agricultural Technology Co., Ltd. and Sowish Biotech Co., Ltd., respectively, supplied the BC and BF used in this investigation. Maize straw was pyrolyzed at a high temperature of 400–450 °C and then dried and put through a 2 mm standard sieve to create granular BC of the same size. BC had a carbon content of 749.3 g kg ^− 1^, 16.0 g kg ^− 1^ N, 3.6 g kg ^− 1^ P, 4.0 g kg ^− 1^ K, a pH of 9.90, and an electrical conductivity of 17.9 mS/cm. The fermentation of maize straw with microbial inoculants such as *B.licheniformis*, *B.gelatinous,* and *S. flavus* led to the production of BF. BF has 450 g kg ^− 1^ of organic matter, 2 billion kg ^− 1^ of active bacteria, 28 g kg ^− 1^ N, 53 g kg ^− 1^ P, and 6 g kg ^− 1^ K.

This experiment used a two-factor design with three replications. 4 N input levels (8.4, 22.5, 45 kg, and 67.5 kg ha ^− 1^) referred to as N0, N1, N2, and N3 were paired with various organic treatments, including BC (150 kg ha ^− 1^), BF (300 kg ha ^− 1^), and without organic amendments (CK). Furthermore, all the fertilizers were applied as base fertilizers for soybean by the hole-apply method. This experiment adjusted chemical fertilizer application as described in (Table 1) to balance the total N, P, and K input in each treatment. Utilizing the inter-maize row ditching fertilization method, 120.0 kg ha^− 1^ N, 105.0 kg ha^− 1^ P_2_O_5_, and 112.5 kg ha^− 1^ K_2_O were applied as base fertilizer for maize, 120.0 kg ha^-1^ N was applied as topdressing during the booting stage.

**Table 1:** The amount of fertilizer applied in this experiment

Organic material	Nitrogen level	N (kg ha^−1^)	P_2_O_5_ (kg ha^− 1^)	K_2_O (kg ha^− 1^)
CK	N0	8.4	63	52.5
	N1	22.5	63	52.5
	N2	45	63	52.5
	N3	67.5	63	52.5
BC	N0	6	55.5	51.6
	N1	20.1	55.5	51.6
	N2	42.6	55.5	51.6
	N3	65.1	55.5	51.6
BF	N0	0	62.73	49.2
	N1	14.1	62.73	49.2
	N2	36.6	62.73	49.2
	N3	59.1	62.73	49.2

Note: Nitrogen application treatment: N0, 8.4 kg ha ^−1^ N; N1, 22.5 kg ha ^−1^ N; N2, 45 kg ha ^−1^ N; N3, 67.5 kg ha ^− 1^ N. Fertilizer treatment: CK, no fertilizer control; BC, biochar; BF, biofertilizer

IS was planted by the wide-narrow row planting with alternating stripes of maize and soybean, as described in the previous study [20]. The distance between maize and soybean strips was 60 cm, row space was 40 cm, and two crop strips with a total width of 2 m. The area of the experiment plot was 36 m^2^, 6 m in length, and 6 m in width, which included three maize-soybean strips. The seed space of maize is 17 cm, and the seed space of soybeans is 8.5 cm. The plant densities of maize and soybean were 58,500 plants ha ^− 1^ and 117,000 plants ha ^− 1^, respectively. There was no strawed retention in the field after harvesting the soybean.

### Soybean biomass and nitrogen partition

At the blooming flower stage (R2), beginning seed stage (R5), and full maturity stage (R8), three comparable soybean plants from each field were sampled. Retrieve the aboveground portion of the soybean above the cotyledon scar, then separate it into the stem, leaf, pod husk, and grains. After 4 days of drying the samples at 80 °C, weigh the organs. Next, employ an automatic Kjeldahl device to measure the nitrogen concentration using the Kjeldahl analytical method (KJeltecTM 8400; FOSS, Hillerod, Denmark). Nitrogen accumulation in different organs is obtained by multiplying the organ’s dry weight and nitrogen content.

### Soybean nodule phenotype and nitrogen fixation ability

At the R2 and R5 stages, soybean roots and nodules in the soil were collected to investigate the nodule number and fresh nodule weight. The sampled areas were 17 cm in width, 80 cm in length, and 20 cm in depth and contained four adjacent soybean plants. Peeled the nodules on the roots and soil, then washed them in tap water. After wiping the water off the nodules with absorbent paper, the number of nodules was counted, and the weight of the fresh nodules was measured. A simultaneous sample of soybean nodules was taken to assess the activity of the nitrogenase enzyme.

The nitrogenase (NA) enzyme activity was assessed using the acetylene reduction assay (ARA) technique [10]. The gas samples were analyzed using gas chromatography-mass spectrometry (GCMS-TQ8040; SHIMADZU, China). The amount of ethylene produced by root nodules per unit of fresh weight (g) in a certain amount of time is used to express the activity of NA (umol^− 1^·g^− 1^ h^− 1^). The nitrogen fixation potential (NFP, mol^− 1^·h^− 1^ ha^− 1^) is obtained by multiplying the NA and fresh nodule weight per ha.

### Soil fertilizer characters

At the R5 and R8 stages, the soil samples were collected using a 2.5 cm diameter auger in a plow layer (0–20 cm). Drill holes in the center of the soybean plants, the soybean rows, and the maize-soybean row to collect soil samples. Soil samples were air-dried, ground, and sieved with 2 mm mesh before being placed in tiny bags.

The enzyme assay kit evaluated the enzyme activity of soil urease and nitrate reductase (Solarbio, Beijing, China). Calculate soil organic material (SOM) by determining the amount of soil organic carbon using the potassium dichromate oxidation method (K_2_Cr_2_O_7_-H_2_SO_4_ oxidation) [18]. The total N content of the soil was determined using Kjeldahl analytical techniques. The alkaline decomposition and diffusion method determined the alkaline hydrolyzable N (AH-N). The molybdenum antimony anti-colorimetry and flame photometry methods were used to measure the Olsen-P and exchangeable K of the soil, respectively [21].

### Soybean yield and yield components

At the R8 stage, 6 m non-sampling strips were harvested to record the soybean grain yield, and the water content was about 13.5%.

### Statistical analysis

The two-factor analysis of variance (ANOVA) and Fisher’s Least Significant Difference (LSD) tests was used to assess the data. Using Origin pro-2022 (Learning version), data analysis and figure drawing were performed (Origin Lab., Hampton, MA, United States).

## Results

### Soybean grain yield and biomass

**Fig. 1:**
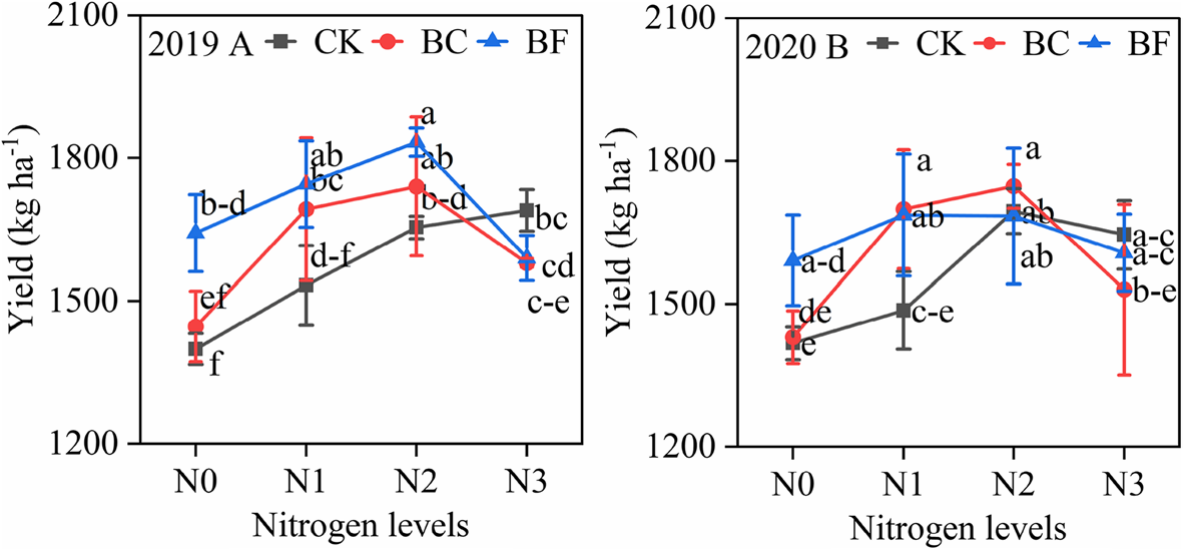
Soybean grain yield under different organic material and nitrogen levels. Note: Fig. 2019 A and 2020 B represent the soybean aboveground dry weight in the 2019 and 2020 growing seasons. R2, R5, and R8 stages represent the blooming flower stage, beginning seeds stage, and fully mature stage of soybean. Nitrogen treatment N0, N1, N2, and N3 represent 8.4 kg ha ^− 1^, 22.5 kg ha ^− 1^, 45 kg ha ^− 1^, and 67.5 kg ha ^− 1^ N, respectively. Fertilizer treatment CK, BC, and BF represent no fertilizer control, biochar, and biofertilizer. The point and error bars represent the mean and standard deviation of the three-replicate data. Different lowercase letters indicated a significant difference between treatments at 0.05 (*p* < 0.05) probability levels

**Fig. 2:**
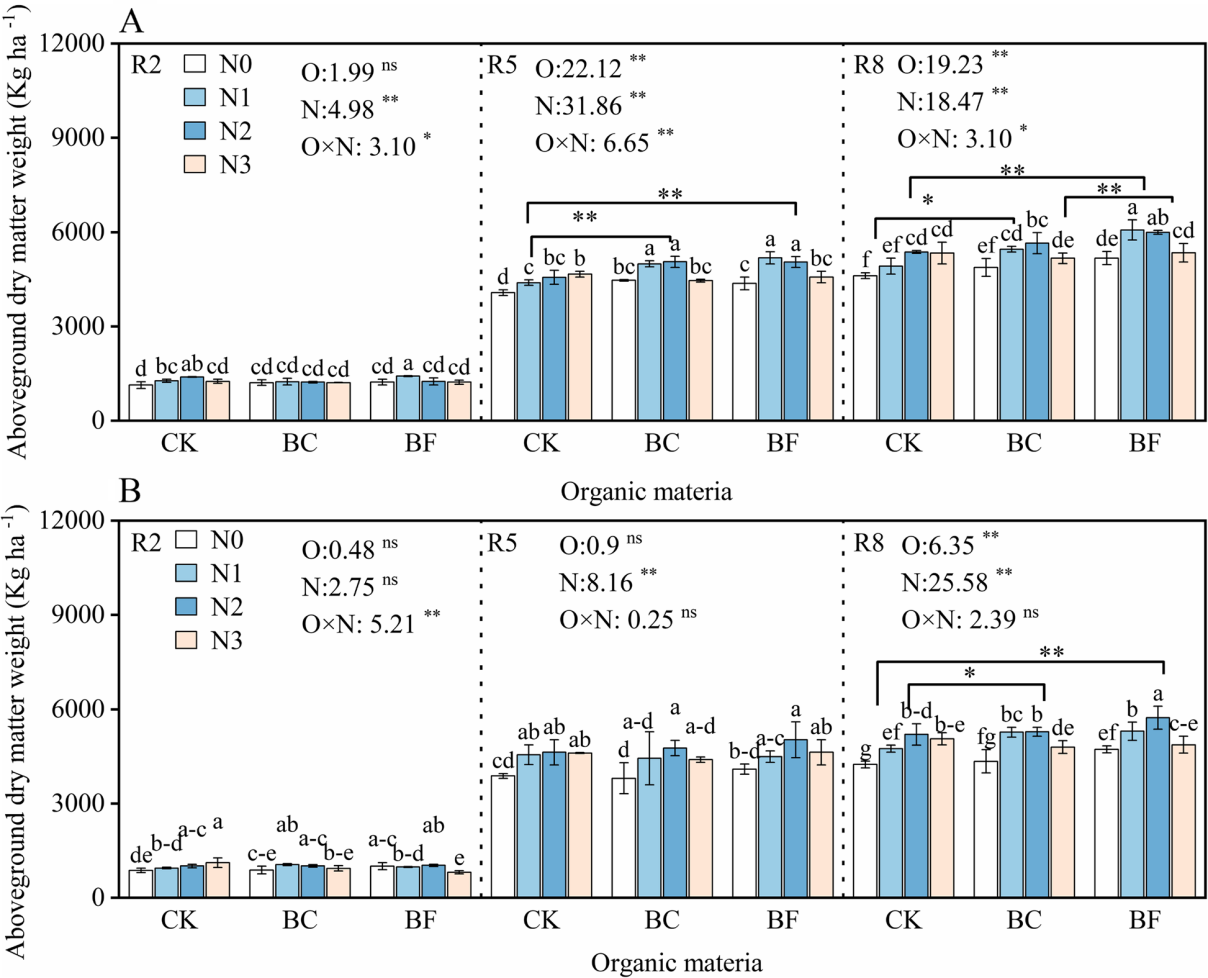
Soybean biomass at the R8 stage under different organic material and nitrogen levels. Note: Fig. A and B represent the soybean aboveground dry weight in the 2019 and 2020 growing seasons, respectively. R2, R5, and R8 stages represent the blooming flower stage, beginning seeds stage, and fully mature stage of soybean. Nitrogen treatment N0, N1, N2, and N3 represent 8.4 kg ha ^− 1^, 22.5 kg ha ^− 1^, 45 kg ha ^− 1^, and 67.5 kg ha ^− 1^ N, respectively. Fertilizer treatment CK, BC, and BF represent no fertilizer control, biochar, and biofertilizer. The error bars represent the mean and standard deviation of the three-replicate data. Different lowercase letters indicated a significant difference between treatments at 0.05 (*p* < 0.05) probability levels. * and ** indicated statistical significance at 0.05 (*p* < 0.05) and 0.01 (*p* < 0.01) probability levels

In 2 years, compared with CK, BC and BF increased soybean yield by 5.2–8.5% and 2.6–2.9%, respectively (Fig. 1). And in N2, BC and BF had the highest grain yields in 2019 and 2020, respectively. Furthermore, under BC and BF, there was no discernible change in grain yield between N1 and N2. The changes in soybean biomass revealed that BC and BF raised soybean biomass prior to the N levels reaching N3 levels (Fig. 2). In 2 years, the highest values of soybean biomass peaking among the N1 and N2, and those values in BF were higher than those in BC and CK. Additionally, compared to CK, soybean biomass increased by 2.3–4.5% and 7.2–11.6% in BC and BF, respectively.

### Nitrogen accumulation and distribution

**Fig. 3:**
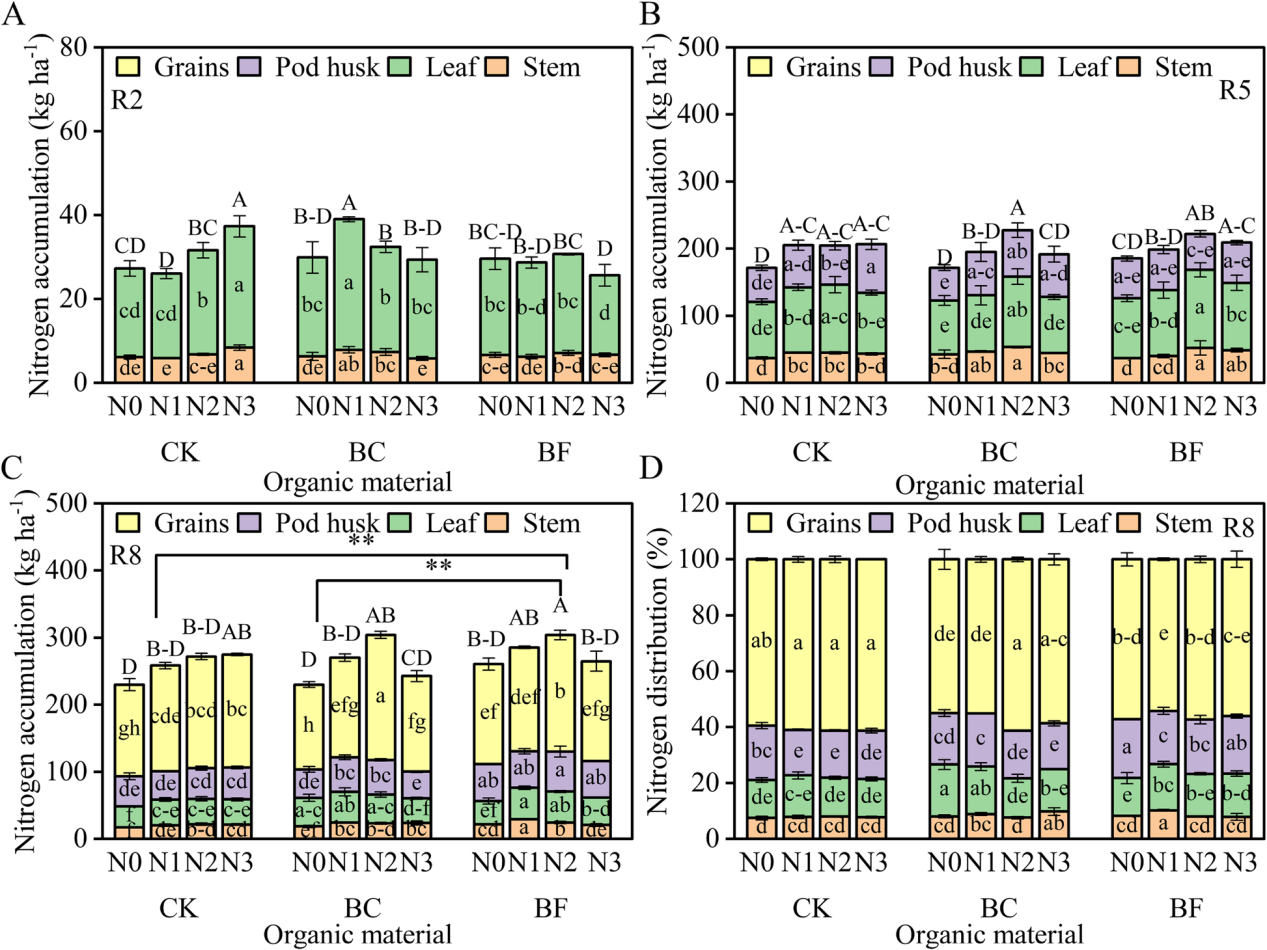
Soybean nitrogen accumulation and distribution at the R8 stage under different organic material and nitrogen levels in 2020. Note Fig. A, B, and C represent the soybean aboveground dry weight at the R2, R5, and R8 stages. Fig. D represents the N distribution in soybean organs at the R8 stage. The growth stage, nitrogen treatment, fertilizer treatment, and significant signs are described in Fig. 2

After the R2 stage, soybean N accumulation quickly increased and peaked at the R8 stage (Fig. 3). While soybean N accumulation under BC and BF was highest in N2, the maximum value of soybean under CK emerged in N3. Compared to CK, BC and BF considerably (*p* < 0.01) increased the nitrogen buildup by 5.1 and 7.7%, respectively. However, the nitrogen distribution analysis revealed that BC and BF reduced the nitrogen distribution ratio in grains compared to CK. Between each treatment, there was no difference in the soybean N accumulation in grains.

### Nodule formation and nitrogen fixation potential

**Fig. 4:**
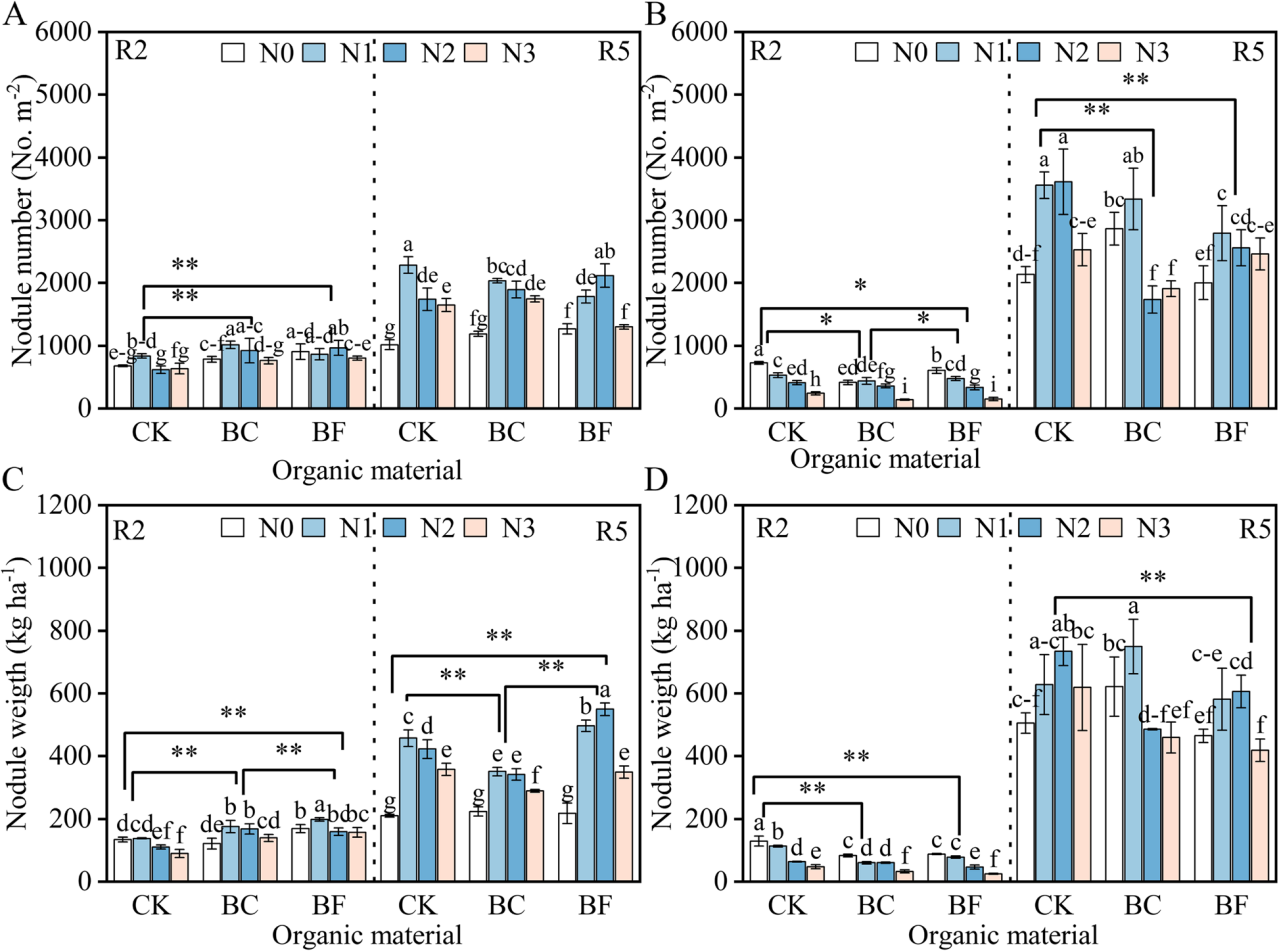
Soybean nodule number and nodule weight under different organic material and nitrogen levels. Note: Fig. A and B represent soybean nodule numbers in 2019 and 2020, respectively. Fig. C and D represent soybean nodule weight in 2019 and 2020, respectively. The growth stage, nitrogen treatment, fertilizer treatment, and significant signs are described in Fig. 2

**Fig. 5:**
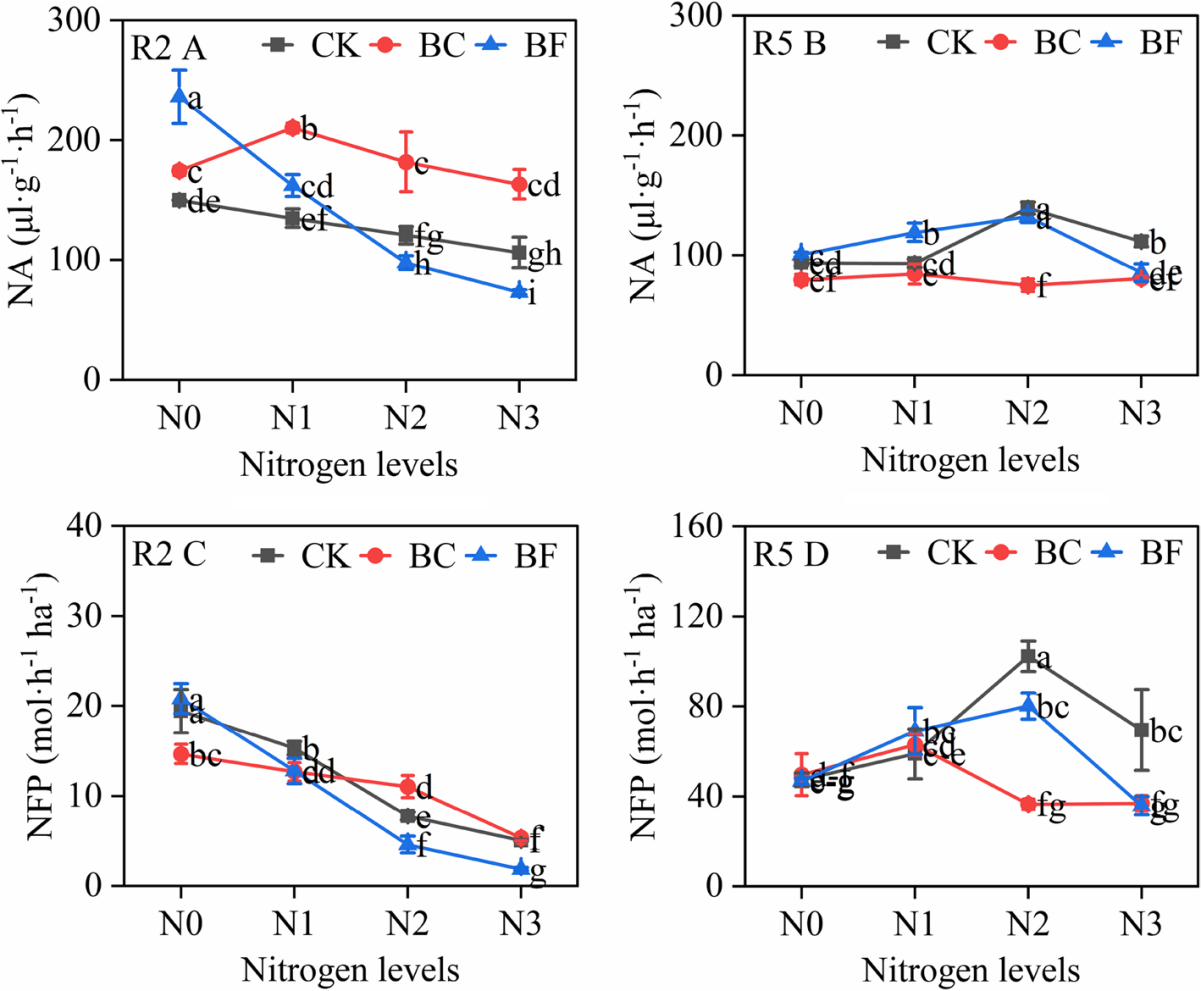
Soybean nitrogenase activity and nitrogen fixation potential under different organic material and nitrogen levels in 2020. Note: Fig. A and B represent soybean nitrogenase activity, and Fig. C and D represent the soybean nitrogen fixation potential at the R2 and R5 stages, respectively. NA: nitrogenase activity; NFP: nitrogen fixation potential. The growth stage, nitrogen treatment, fertilizer treatment, and significant signs are described in Fig. 1

At the R5 stage, soybean nodule number and weight were highest in N2 (Fig. 4). The influence of BC and BF on soybean nodulation over 2 years revealed opposing tendencies, with BC and BF increasing the number of nodules and nodule weight in 2019 whereas these values dropped in 2020. BF had a much higher number and weight of soybean nodules than BC. The values of NA at the R2 stage were higher than that at the R5 stage, and the change in NFP showed opposite trends (Fig. 5). The highest NA and NFP values at the R5 stage were found in N2, while those values in BC and BF were lower than in CK. Additionally, the NA and NFP values under BF were higher than those under BC for each N level.

### Soil fertilizer characters analysis

**Fig. 6:**
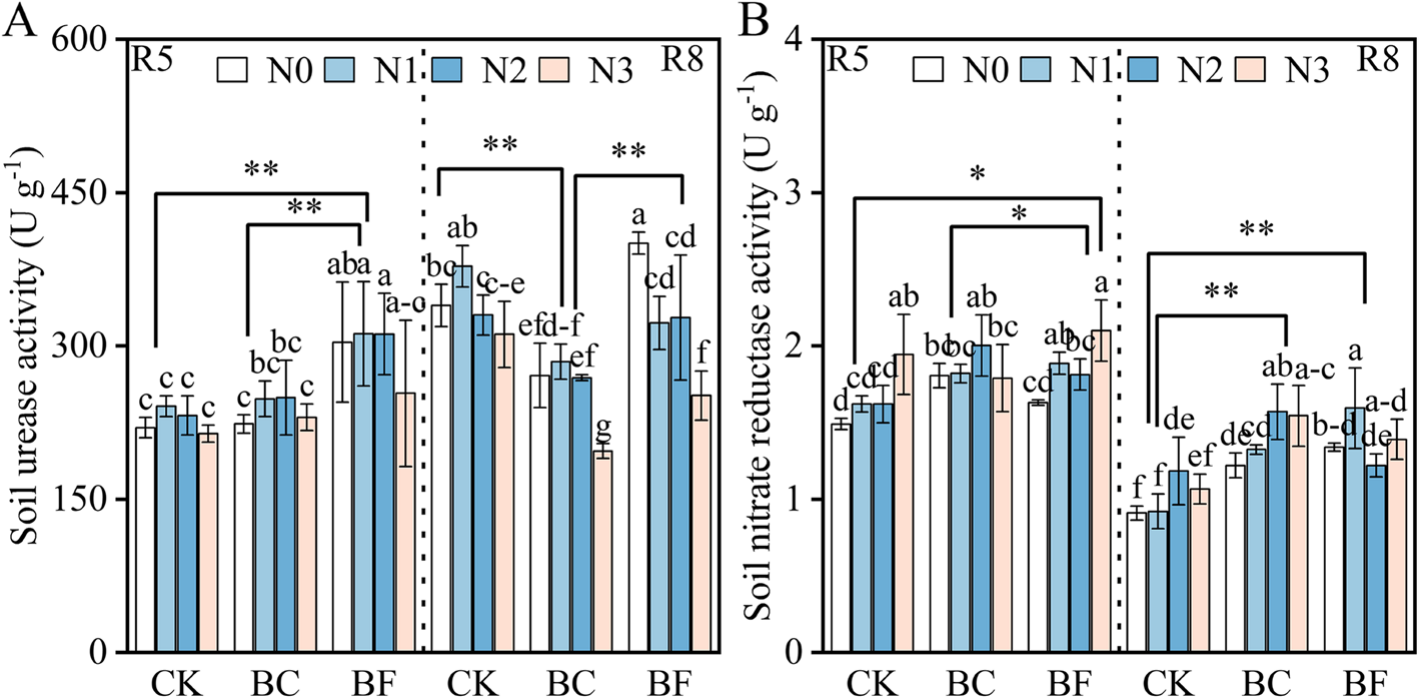
Soil urease activity and soil nitrate reductase activity under different organic material and nitrogen levels. Note: Fig. A and B represent soil urease activity and soil nitrate reductase activity, respectively. The growth stage, nitrogen treatment, fertilizer treatment, and significant signs same as described in Fig. 2

At the R5 and R8 stages, BF had considerably (*p* < 0.01) stronger soil urease and nitrate reductase activity than CK (Fig. 6). Wh﻿en com﻿pa﻿red to CK at the R8 stage, BC decreased soil urease and enhanced nitrate reductase acti﻿vit﻿y. According to the results of the soil properties, total N, SOM, Olsen-P, and AH-N values i﻿n BC a﻿nd BF rose in comparison to CK, and those values in BF were greater than in BC (Table 2). Total N, SOM, Olsen-P, and AH-N concentrations were considerably (*p* < 0.05) higher in BF than in CK, increasing by 13.0, 17.1, 22.20, and 7.4%, respectively. The difference in total N and SOM between BC and BF was negligible. The application of BC and BF did not significantly affect the exchangeable K of the soil.

**Table 2:** Effect of organic material and nitrogen application level on soil properties

Organic material	Nitrogen level	Total N (g kg ^− 1^)	SOM (g kg^− 1^)	Olsen- P (mg kg^− 1^)	Exchangeable K (mg kg^− 1^)	Alkali-hydrolysable N (mg kg^− 1^)
CK	N0	1.27 ± 0.02e	23.63 ± 1.12f	18.88 ± 3.51b	164.33 ± 38.63ab	49.46 ± 3.25ab
	N1	1.35 ± 0.09b-e	24.41 ± 1.91ef	33.07 ± 10.98ab	177.33 ± 35.23ab	52.34 ± 3ab
	N2	1.29 ± 0.1de	25.42 ± 0.7c-f	23.86 ± 2.39ab	156 ± 33.06b	54.74 ± 4.01ab
	N3	1.31 ± 0.12c-e	24.26 ± 0.82ef	32 ± 22.47ab	159 ± 20.81b	50.78 ± 2.55ab
	mean	1.31 ± 0.08B	24.53 ± 1.26C	26.95 ± 12.43B	164.17 ± 29.11A	51.93 ± 3.51A
BC	N0	1.48 ± 0.04a-c	27.67 ± 2.38b-d	20.36 ± 3.25b	161.67 ± 12.5ab	52.58 ± 4.99ab
	N1	1.54 ± 0.14a	28.04 ± 1.98a-c	29.53 ± 4.64ab	188.67 ± 26.08ab	52.58 ± 5.04ab
	N2	1.48 ± 0.05a-c	26.96 ± 1.28b-e	20.48 ± 2.9b	202 ± 14.8ab	45.86 ± 3.97bc
	N3	1.43 ± 0.02a-e	24.75 ± 1.36d-f	21.68 ± 1.91ab	241 ± 55.33a	42.42 ± 6.54c
	mean	1.49 ± 0.08A	27.05 ± 1.98B	23.01 ± 4.87AB	198.33 ± 40.51A	48.36 ± 7.56A
BF	N0	1.47 ± 0.13a-d	28.56 ± 0.3ab	32.54 ± 8.17ab	202.33 ± 69.76ab	51.62 ± 7ab
	N1	1.53 ± 0.25ab	28.21 ± 2.23ab	37.93 ± 14.15a	210.67 ± 75.08ab	56.18 ± 2.04a
	N2	1.49 ± 0.03a-c	27.62 ± 0.66b-d	35.86 ± 16.12ab	212 ± 84.66ab	54.98 ± 5.41ab
	N3	1.42 ± 0.1a-e	30.5 ± 2.2a	21.08 ± 2.97ab	160.33 ± 31.82b	55.94 ± 11.84a
	mean	1.48a ± 0.14A	28.72 ± 1.77A	32.83 ± 12.02A	196.33 ± 62.32A	54.55 ± 6.92A
F-value test						
Organic material (O)		8.9**	18.68**	2.09	1.96	4.64**
N application (N)		1.17	0.08	1.56	0.2	1.13
O*N		0.21	2.07	0.8	1.08	1.79

Note: Nitrogen application treatment: N0, 8.4 kg ha ^−1^ N; N1, 22.5 kg ha ^−1^ N; N2, 45 kg ha ^−1^ N; N3, 67.5 kg ha ^−1^ N. CK: no fertilizer control; BC: biochar; BF: biofertilizer; SOM: soil organic material; Olsen-P: Olsen- phosphorus; K: potassium. Different lowercase letters in the same column in each growth year indicated a significant difference between each treatment at 0.05 probability levels. The different capital letters in the same column in each growth year indicate a significantly different at 0.05 probability levels. * and ** indicated statistical significance at 0.05 (*p* < 0.05) and 0.01 (*p* < 0.01) probability levels

## Discussion

This experiment showed that using BF in combination with N2 is a potential strategy to maintain stable yield production with the reduced N input, increasing soybean yield by 5.2–8.5% when the total N input was lowered by around 38.5% (Fig. 1). The findings indicating the maximum soybean grain yield occurred in N3 under CK was in line with earlier researcher [12], who demonstrated that the 67.5 kg ha ^− 1^ N input for soybean is suited for low N input and high yield output in IS. Moreover, the findings showing BC and BF had a favorable impact on the creation of soybean yield in N2 may be seen in the alteration in the accumulation and distribution of biomass and N in various organs.

Additionally, this study discovered that BC and BF boosted soybean total N accumulation while decreasing the distribution ratio in grains, indicating that the increase in soybean yield was caused by the increase in total biomass rather than the transfer efficiency of biomass to grains (Figs. 2 and 3). Previous studies have proved that the high yield crops were characterized by high biomass accumulation rates and high transport ratio [22]. As a result, the rise in total biomass and buildup of nitrogen in soybeans may somewhat offset the detrimental effects of the decline in the distribution ratio in grains. Similar findings were also reported that increasing the whole-plant biomass increases grain production [23]. The fact that the soybean biomass and N accumulation values in BF were considerably (*p* < 0.05) higher than in BC may help to explain why BF’s yield increase was greater than BC’s.

Under low N input or low soil fertilizer circumstances, the N from the BNF plays a crucial role in increasing the ability of soybean to generate yield [24]. The findings showed that the number and weight of soybean nodules increased with low nitrogen input (N1 and N2) and reduced with high nitrogen input (N3) were in line with earlier findings that the nodulation is constantly suppressed by high nitrogen availability, or the N inhibitory effect [25, 26]. This investigation revealed that BC and BF decrease soybean nodule number, nodule weight, NA, and NFP, in contrast to the increase in N accumulation in BC and BF, which shows that BC and BF suppressed soybean BNF in N1 and N2 levels (Figs. 4 and 5).

The previous researchers’ findings were different from this experiment. They have demonstrated that the BF greatly encouraged soybean nodulation and BNF in different legume crops [27, 28]. Meanwhile, there is less study on the direct impact of BC on soybean BNF. Most studies focus on BC’s impact on soil characteristics and nutrient availability [29, 30]. Some research uses BC inoculation with bacteria to look at changes in soybean nodulation [31]. Compared to BC, BF had a larger NFP which may have contributed to BF’s higher biomass and N accumulation, suggesting that BC had a more detrimental impact on soybean BNF.

Previous research has demonstrated that the soybean has two primary sources of nitrogen: inorganic nitrogen absorbed by the roots and organic nitrogen produced by the BNF of root nodules [23]. The conclusion drawn from this experiment is that BC and BF helped the soybean receive N from the soil rather than the BNF. This is supported by the finding that BC and NF inhibit soybean BNF but boost N accumulation. Numerous studies have shown that the application of organic amendment increases the nutrients available in the soil by boosting the soil’s physical and chemical characteristics and fertility [32] promoting the soil nutrient cycle [33], and speeding up soil ventilation [34].

The concentrations of total N, SOM, AH-N, Olsen-P, and exchangeable K in soil were enhanced by BC and BF, particularly BF, in this study (Table 2). Changes in the soil’s total N, SOM, AH-N, and Olsen-P reflect the ability of soil to provide nutrients for crop growth and development [35]. The result that BF enhanced soil nutrient content was consistent with previous research that demonstrated that BF increases the soil’s content of organic carbon, cation exchange capacity, and nitrogen and phosphorus availability [27, 34]. Additionally, the rise in soil N transformation-related enzymes, such as urease and nitrate reductase during the R5 and R8 stage, was greater in BF than in other regions, which may help to partially explain why BF had the greatest levels of total N and AH-N. The conclusion that BC and BF treatment enhances soybean’s ability to absorb N from the soil and inhibits soybean nodulation and BNF may be supported by study results showing that BC and BF enhanced the availability of soil nutrients.

Furthermore, the availability of soil nutrients not only affects soybean nodulation but is a crucial factor in determining whether an agricultural system can sustain long-term high-yield output [36]. In order to achieve high-yield production without compromising soil quality and nutrient recycling, sustainable agriculture patterns must be used [37]. The experiment’s findings showed that BC and BF boosted soybean production and soil nutrient availability in N2 levels may also imply that using BC and BF in IS with minimal N inputs could be a viable strategy for sustainable agricultural development. Further testing of this methodology is required while the mechanism of BC and BF on soybean BNF and soil characteristics are investigated.

## Conclusion

Applying BC or BF in conjunction with the proper fertilizers is a great way to increase crop yields quickly while preserving sustainability over the long term in IS. Although both BC and BF suppressed soybean nodulation and BNF, BF significantly increased biomass and N accumulation, leading to the maximum grain yield in N2 under BF. When it comes to enhancing the availability of soil nutrients, including total N, SOM, Olsen-P, and Akali-hydrolysable N, BF outperforms BC. In conclusion, applying BF with N2 (45 kg ha ^− 1^ N) boosted soybean production favorably and kept soil productivity in IS. Additionally, more research is still needed to determine how BC and BF control soybean nodulation and N accumulation under varied planting patterns and soil fertilizer conditions.

## Data Availability

The original contributions presented in the study are included in this article. Further inquiries can be directed to the corresponding author.

## Abbreviations

AH-N: alkaline hydrolyzable N
ARA: acetylene reduction assay
BC: biochar
BF: biofertilizer
NA: nitrogenase activity
BNF: biological nitrogen fixation
NFP: nitrogen fixation potential
N: nitrogen
Olsen-P: Olsen- phosphorus
OA: organic amendment
R2: blooming flower stage
R5: beginning seed stage
R8: full maturity stage
SOM: soil organic material
K: potassium
IS: Maize soybean relay strip intercropping system.
